# Metal ions and redox balance regulate distinct amyloid-like aggregation pathways of GAPR-1

**DOI:** 10.1038/s41598-019-51232-7

**Published:** 2019-10-21

**Authors:** Jie Sheng, Nick K. Olrichs, Willie J. Geerts, Dora V. Kaloyanova, J. Bernd Helms

**Affiliations:** 1 Department of Biochemistry and Cell Biology, Faculty of Veterinary Medicine, Utrecht University, Utrecht, The Netherlands; 2 Biomolecular Imaging, Bijvoet Center, Utrecht University, Utrecht, The Netherlands

**Keywords:** Biochemistry, Protein folding, Protein aggregation

## Abstract

Members of the CAP superfamily (Cysteine-rich secretory proteins, Antigen 5, and Pathogenesis-Related 1 proteins) are characterized by the presence of a structurally conserved CAP domain. The common structure-function relationship of this domain is still poorly understood. In this study, we unravel specific molecular mechanisms modulating the quaternary structure of the mammalian CAP protein GAPR-1 (Golgi-Associated plant Pathogenesis-Related protein 1). Copper ions are shown to induce a distinct amyloid-like aggregation pathway of GAPR-1 in the presence of heparin. This involves an immediate shift from native multimers to monomers which are prone to form amyloid-like fibrils. The Cu^2+^-induced aggregation pathway is independent of a conserved metal-binding site and involves the formation of disulfide bonds during the nucleation process. The elongation process occurs independently of the presence of Cu^2+^ ions, and amyloid-like aggregation can proceed under oxidative conditions. In contrast, the Zn^2+^-dependent aggregation pathway was found to be independent of cysteines and was reversible upon removal of Zn^2+^ ions. Together, our results provide insight into the regulation of the quaternary structure of GAPR-1 by metal ions and redox homeostasis with potential implications for regulatory mechanisms of other CAP proteins.

## Introduction

Golgi-Associated plant Pathogenesis-Related protein (GAPR-1) is a mammalian protein which belongs to the CAP (Cysteine-rich secretory proteins, Antigen 5, and Pathogenesis-related 1 proteins) superfamily of proteins^1^. All members of the CAP superfamily contain a structurally conserved CAP domain containing four conserved CAP motifs (CAP1-4). The CAP domain tertiary structure has a unique α-β-α sandwich fold in which α-helices flank a central antiparallel β-sheet^1^. The CAP domain also contains a conserved central cavity formed by two histidine residues and two acidic amino acids, usually glutamic acid^1^. Several CAP superfamily members have been shown to bind different metal ions via this conserved central cavity^2–7^. Most CAP proteins have an N-terminal signal peptide for secretion into the extracellular cell environment where they exhibit endo- or paracrine functions^1^. The presence of a variety of additional domains at the C-terminal or sometimes N-terminal end of the CAP domain underlies the high functional diversity within the CAP superfamily^1,8^. Certain specific functions have been attributed to the CAP domain, such as protease activity^9^ as well as lipid and sterol binding and transport^8,10,11^. However, a common functionality for the CAP domain is still unknown.

GAPR-1 is the most ancient CAP subfamily member in vertebrates that evolved from the CAP/PR domain in bacteria^1,12^. These one-domain proteins evolved in invertebrates and mammals into proteins with two domains, connected via a hinge region. GAPR-1 does not contain a hinge region or any other domain. Hence, GAPR-1 consists almost exclusively of a CAP domain, making it a suitable model protein to study the structure-function relationship of this domain^13^. GAPR-1 functions as a negative regulator of autophagy in mammalian cells^14^. It is associated with lipid-enriched microdomains at the cytosolic leaflet of Golgi membranes where it inhibits autophagy by anchoring the autophagy-inducing protein Beclin 1 to the membrane^14,15^. GAPR-1 is also involved in the type I interferon signaling pathway in response to Toll-like receptor 4^16^. At a structural level, GAPR-1 forms homodimers on Golgi membranes and crystallizes as a dimer^17^. Binding of inositol-hexakisphosphate (IP6) induces an alternative dimer conformation, with one of the monomers rotated by 28.5 degrees^18^. Recently, a GAPR-1 mutant, which is defective in interacting with Beclin 1, was shown to adopt yet another dimer conformation^19^. Upon interaction with phosphatidylinositol- and cholesterol-containing liposomes, GAPR-1 forms oligomers and amyloid-like structures^20^. This indicates that structural dynamics play an important role in modulating GAPR-1 function. The physiological and molecular mechanisms underlying these structural dynamics are still poorly understood. The presence of potentially amyloidogenic sequences in the CAP1 and CAP2 motifs^20^ led us to propose the CAP domain as a structural domain, regulating protein-protein interactions via its amyloidogenic properties^13^.

Metal homeostasis is frequently involved in the regulation of protein conformational dynamics^21–23^. We recently showed that Zn^2+^ binding to GAPR-1 regulates the formation of amyloid-like structures upon interaction with heparin *in vitro*^24^. In this study, we demonstrate that Cu^2+^ induces GAPR-1 fibrillation in the presence of heparin through an alternative pathway. Unlike zinc, copper ions exert their effect independently of the conserved putative metal binding site, but through oxidation of the two cysteine residues in the formation of disulfide-linked oligomeric intermediates. This infers a critical role for redox and metal homeostasis in the regulation of specific high-molecular weight assemblies of GAPR-1.

## Results

### Copper ions induce alternative GAPR-1 amyloid-like aggregation

**Figure 1:**
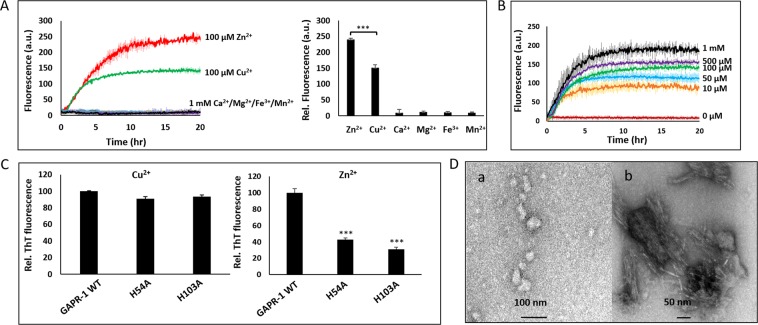
Cu^2+^ induces alternative GAPR-1 amyloid-like aggregation. (**A**) Kinetics of ThT fluorescence enhancement of 15 μM GAPR-1 incubated with 37.5 μM heparin and 100 μM Zn^2+^/100 μM Cu^2+^/1 mM Ca^2+^/1 mM Mg^2+^/1 mM Fe^3+^/1 mM Mn^2+^ at 37 °C. The results represent the means (line in bold +/− S.D.) of three independent experiments. ThT fluorescence intensity of each reaction after 20 h incubation is shown in the right panel. Stars indicate the statistical significance using t-test (two-sample assuming equal variances): ***P-value = 0.0004 < 0.001. (**B**) Kinetics of ThT fluorescence enhancement of 15 μM GAPR-1 incubated with 37.5 μM heparin in the presence of increasing concentrations of Cu^2+^ (0–1,000 μM) at 37 °C. The results represent the means (line in bold +/− S.D.) of three independent experiments.(**C**) 15 μM GAPR-1 WT, H54A or H103A was incubated with 5 μM heparin and 100 μM Cu^2+^ (left panel) or Zn^2+^ (right panel) at 37 °C. ThT fluorescence intensity after 20 h incubation is shown for the histidine mutants relative to that of WT GAPR-1 for each metal ion, respectively. The results represent the means (+/− S.D.) of three independent experiments. A difference between Cu^2+^ and Zn^2+^ induced amyloid formation is also observed at other heparin concentrations. Stars indicate the statistical significance using t-test (two-sample assuming equal variances): ***P-value < 0.001 (P_H54A_ = 0.0002 and P_H103A_ = 0.0001). (**D**) Transmission electron micrographs of 15 μM GAPR-1 following incubation with 37.5 μM heparin and 100 μM Cu^2+^ at 37 °C for 3 h (a) and 24 h (b). Scale bars represent 100 nm in panel a and 50 nm in panel b.

GAPR-1 forms amyloid-like structures when incubated with heparin in the presence of zinc ions^24^. Several CAP superfamily members have been reported to bind zinc and other metal ions at a highly conserved putative binding site in the CAP domain^2–7^. In order to determine whether the modulation of GAPR-1 amyloidogenicity is specific for zinc, GAPR-1 was incubated with various metal ions in the presence of heparin and ThT fluorescence was monitored over time. In the presence of Cu^2+^, enhancement of ThT fluorescence intensity was observed. Other metal ions, including Ca^2+^, Mn^2+^, Mg^2+^ and Fe^3+^, had no effect, even at concentrations of 1 mM (Fig. 1A). The increase in ThT fluorescence was Cu^2+^ concentration-dependent (Fig. 1B). Compared to equimolar concentrations of Zn^2+^, the ThT fluorescence increase induced by Cu^2+^ was significantly lower (Fig. 1A). Our previous study had shown that mutations in the putative metal-binding site (H54A and H103A) of the CAP domain impaired zinc-dependent aggregation of GAPR-1^24^ (also shown in Fig. 1C, right panel). To determine whether this site is also involved in copper-induced amyloid-like aggregation, we analyzed the effect of Cu^2+^ on GAPR-1 H54A and GAPR-1 H103A mutant proteins. Surprisingly, copper-induced ThT fluorescence enhancement was not significantly affected in both histidine mutants as compared to WT GAPR-1 (Fig. 1C). These results suggest that copper-dependent initiation of GAPR-1 amyloid formation is mechanistically different from Zn^2+^-induced aggregation. In support of this, structural analysis of the aggregates formed in the presence of Cu^2+^ by TEM showed spherical particles of irregular size and clustered amyloid-like fibrillar structures after 3 and 24 h, respectively (Fig. 1D) that were morphologically markedly different as compared to the Zn^2+^-induced oligomers and amyloid-like aggregates^24^.

### Copper binding induces structural changes in GAPR-1

**Figure 2:**
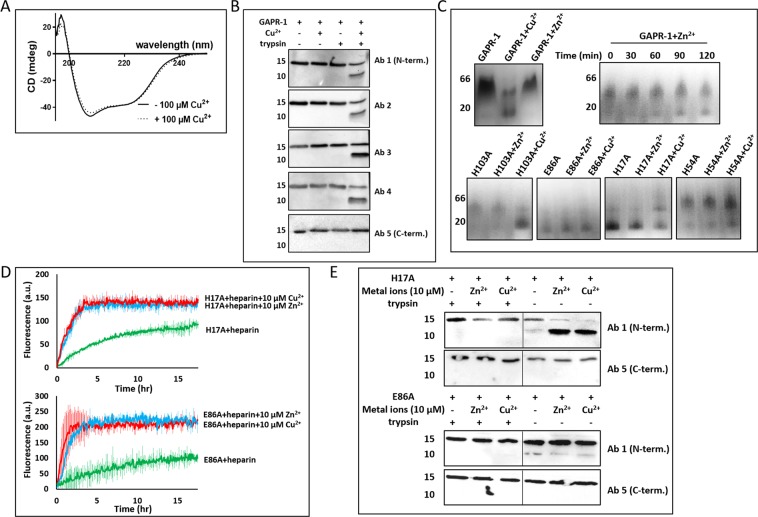
Cu^2+^ binding induces conformational changes in GAPR-1. (**A**) Far-UV CD spectra of 15 μM GAPR-1, recorded in the absence (solid line) and presence (dotted line) of 100 μM Cu^2+^. (**B**) Western blot analysis of limited trypsin digestion of GAPR-1 incubated with Cu^2+^ using five different GAPR-1 antibodies. GAPR-1 was incubated in the absence and presence of 100 μM Cu^2+^ at 37° for 30 min, followed by incubation without or with trypsin in the molar ratio of 1:50 (trypsin: GAPR-1) for 30 min at 37 °C. (**C**) BN-PAGE analysis of GAPR-1 WT (upper panel) and mutants (lower panel) in the absence and presence of metal ions. 2 μg GAPR-1 WT/mutants were incubated with 100 μM Cu^2+^ or Zn^2+^ at room temperature for 5 min (upper left). 2 μg WT GAPR-1 incubated with 100 μM Zn^2+^ for prolonged times as indicated (upper right). (**D**) Kinetics of ThT fluorescence enhancement of 15 μM H17A and E86A, respectively, incubated with 37.5 μM heparin in the absence and presence of 10 μM Zn^2+^ or Cu^2+^ at 37 °C. The results represent the means (line in bold +/− S.D.) of three independent experiments. (**E**) Trypsin susceptibility of monomeric GAPR-1 mutants in the absence or presence of metal ions. Western blot analysis of limited trypsin digestion of H17A/E86A incubated without or with 10 μM Zn^2+^ or Cu^2+^ using N-terminal and C-terminal GAPR-1 antibodies, respectively.

Zn^2+^-binding to GAPR-1 did not induce a detectable change in the secondary structure of GAPR-1, but exposed a trypsin cleavage site, resulting in the trypsin-dependent release of a ~12 kDa C-terminal fragment^24^. Surprisingly, similar results were obtained with Cu^2+^: Copper binding to GAPR-1 did not induce a change in secondary structure as determined by CD spectroscopy (Fig. 2A) but did induce a subtle change in the GAPR-1 structure, yielding a trypsin-sensitive proteolytic product of similar molecular weight as in the conditions with zinc (Fig. 2B). Using antibodies specific for different regions of GAPR-1, the digestion product was also shown to be cleaved at the C-terminal part of the protein (Fig. 2B) suggesting that copper binding causes a similar, if not the same structural change.

Exposure of the trypsin-sensitive cleavage site could be the result of a subtle conformational change and/or a change in the quaternary structure of GAPR-1. Previous studies on plant PR-1 proteins revealed that dimerization was linked directly to protease resistance. Thus, a shift in the monomer/dimer ratio of GAPR-1 could result in exposure of a trypsin cleavage site that is hidden at the dimer interface. To investigate whether metal binding to GAPR-1 affected its quaternary structure, we employed Blue Native PAGE (BN-PAGE). In the absence of metal ions or in the presence of 100 µM Zn^2+^, GAPR-1 migrates as a diffuse band at approximately the dimer-tetramer molecular weight range (Fig. 2C, upper left panel). Following a 5 min incubation with 100 µM Cu^2+^, two well-defined bands were visible at molecular weights corresponding to GAPR-1 dimers and monomers (Fig. 2C, upper left). This suggests that the quaternary structure of GAPR-1 is destabilized by copper binding, resulting in the generation of monomeric species. In marked contrast, Zn^2+^ also induced a shift to monomeric GAPR-1 species, but only at later time points (Fig. 2C, upper right panel). The GAPR-1 histidine mutants H54A and H103A remained sensitive to Cu^2+^ and formed monomers in the presence of this metal ion (Fig. 2C), in agreement with the results described in Fig. 1C.

To investigate a role for GAPR-1 monomers in the formation of Cu^2+^-induced amyloid-like fibrils, we generated GAPR-1 mutants that are impaired in forming native multimers. A predominantly monomeric protein was observed for mutants E86A and H17A (Fig. 2C, lower panel). The highly conserved Glu86 is located along the crystal dimer interface. A previous study on plant PR-1 proteins showed that mutation of the corresponding glutamate impaired its ability to form native dimers^25^. His17 is another highly conserved residue that is not located near the dimer interface but part of the CAP3 signature motif. Both mutants were analyzed for their capacity to form amyloid-like aggregates in the presence of heparin. Strikingly, both H17A and E86A mutants showed an increase in ThT fluorescence when incubated with heparin in the absence of metal ions (Fig. 2D). In the presence of low concentrations of Zn^2+^ or Cu^2+^ (10 µM), ThT intensity strongly increased. In the absence of heparin no increase in ThT fluorescence was observed under any condition. The monomeric GAPR-1 mutants were analyzed in the limited trypsin digestion assay and both H17A and E86A (Fig. 2E) were sensitive to trypsin in the absence of metal ions. The presence of Zn^2+^ or Cu^2+^ enhanced the proteolytic digestion of GAPR-1 H17A and antibody recognition showed that the ~12 kDa digestion product resulted from cleavage of the C-terminal part of the protein (Fig. 2E). We therefore consider it likely that exposure of the trypsin-sensitive cleavage site is the result of a subtle conformational change in combination with a change in the quaternary structure of GAPR-1.

### Copper-induced fibrillation pathway requires disulfide bond formation

**Figure 3:**
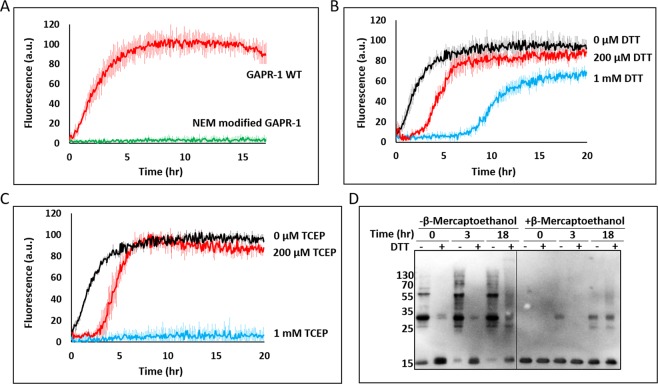
Copper-induced fibrillation pathway requires disulfide bond formation. (**A**) Kinetics of ThT fluorescence enhancement of 15 μM WT GAPR-1 or NEM-modified GAPR-1 incubated with 37.5 μM heparin and 100 μM Cu^2+^ at 37 °C, (**B**) in the additional presence of DTT (0–1,000 μM) (**C**) or TCEP (0–1,000 μM). The results represent the means (line in bold +/− S.D.) of three independent experiments. (**D**) 15 μM GAPR-1 was incubated with 37.5 μM heparin and 100 μM Cu^2+^ in the absence and presence of 1 mM DTT at 37 °C. Aliquots were taken at 0 h, 3 h and 18 h and Laemmli sample buffer with or without BME was added for SDS-PAGE and Western blot analysis was performed using a C-terminal GAPR-1 antibody.

Besides histidines, Cu^2+^ is also known to readily interact with cysteines^26^. GAPR-1 has two cysteines, which are not involved in disulfide bridge formation within GAPR-1 molecules but could, *e.g*., be involved in disulfide bridge formation between GAPR-1 molecules during oligomerization. After pre-incubation of GAPR-1 with N-ethylmaleimide (NEM) to modify cysteine residues, no amyloid-like fibers were observed anymore in the ThT fluorescence assay in the presence of heparin and Cu^2+^ (Fig. 3A). Western blot analysis confirmed the inability of NEM-modified GAPR-1 to form HMW structures in the presence of Cu^2+^ and heparin (Supplementary Fig. 1). To further investigate a role of disulfide bonds in Cu^2+^-mediated amyloid-like aggregation, WT GAPR-1 was incubated with heparin and 100 μM Cu^2+^ under reducing conditions to prevent disulfide bond formation. Strikingly, with increasing concentrations of DTT, a prolonged delay in the onset of ThT fluorescence increase was observed (Fig. 3B). This suggests disulfide bond formation plays a crucial role during the initiation of amyloid formation under reducing conditions. To exclude that fluorescence kinetics were affected by possible interactions between Cu^2+^ and thiol groups of DTT, experiments were repeated using TCEP. A similar delay was observed for 200 μM TCEP, whereas 1 mM TCEP even caused full inhibition over a period of 20 h (Fig. 3C). The inhibition of oligomeric and/or amyloid-like structures under reducing conditions was confirmed by Western blot analysis. In the presence of DTT, the amount of oligomers and HMW structures was drastically lower at each timepoint as compared to incubation in the absence of DTT (i.e., Fig. 3D, −BME). Both in the absence and presence of DTT, oligomeric and HMW structures were observed after 18 h incubation that were resistant to the routinely applied denaturing conditions of SDS-PAGE (Fig. 3D, +BME), consistent with characteristics of amyloid-like fibrils.

### Cysteine residues of GAPR-1 are involved in Cu^2+^ binding

**Figure 4:**
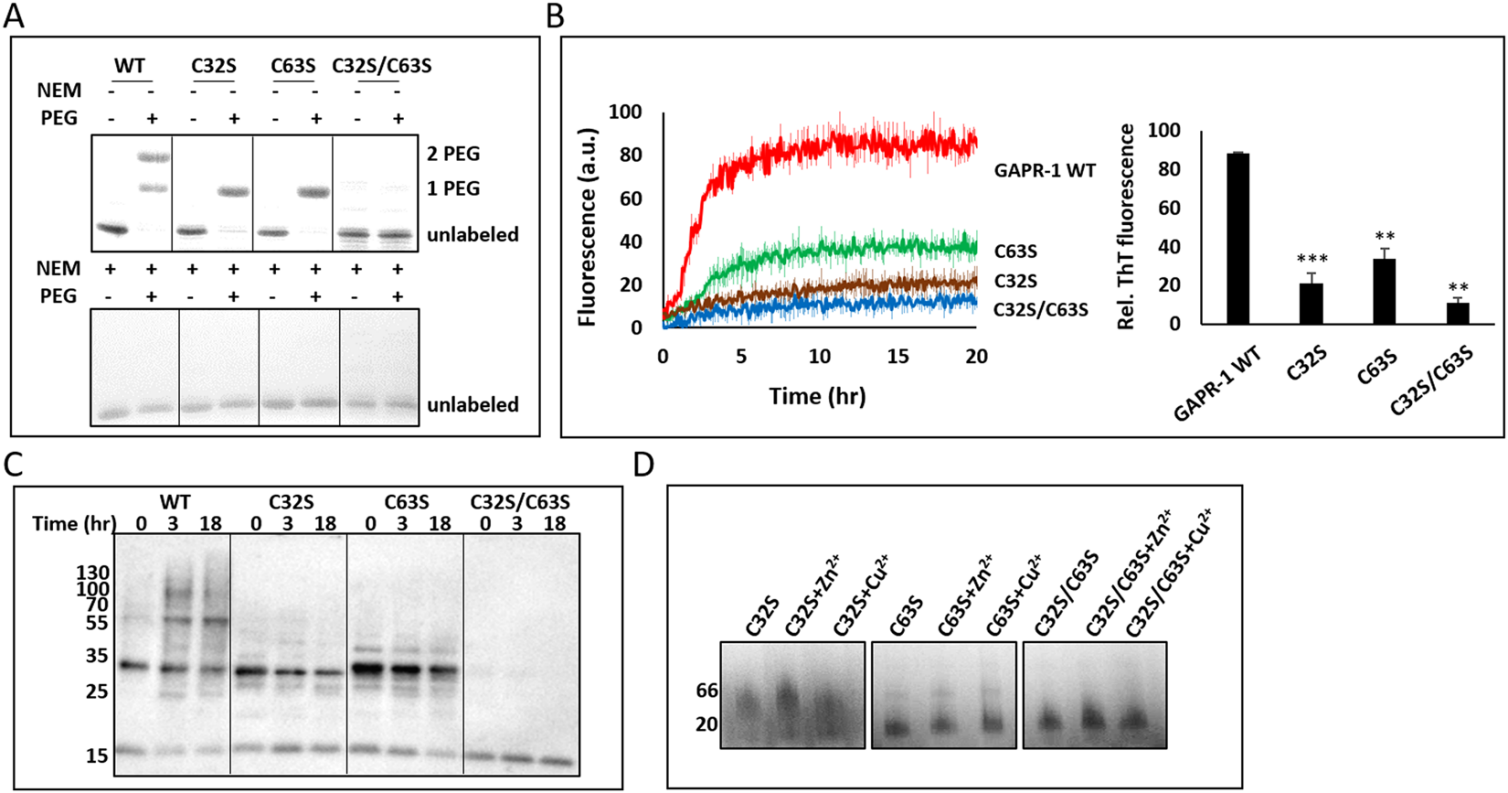
Cysteine residues of GAPR-1 are involved in Cu^2+^ binding. (**A**) PEG-maleimide labeling of cysteines analyzed by SDS-PAGE. 60 μM GAPR-1 WT, C32S, C63S or C32S/C63S was incubated with 1 mM PEG-maleimide at room temperature for 1 h following a pre-incubation with or without 10 mM NEM at room temperature for 1 h. (**B**) Kinetics of ThT fluorescence enhancement of 15 μM GAPR-1 WT, C32S, C63S or C32S/C63S incubated with 37.5 μM heparin and 20 μM Cu^2+^ at 37 °C (left panel). ThT fluorescence intensity of the cysteine mutants after 20 h incubation relative to WT GAPR-1 (right panel). The results represent the means (line in bold +/− S.D.) of three independent experiments. Similar results were obtained at 100 μM Cu^2+^. Stars indicate the statistical significance using t-test (two-sample assuming equal variances): **0.001 < P-value < 0.01; ***P-value < 0.001 (P_C32S_ = 0.0002, P_C63S_ = 0.0017, P_C32S/C63S_ = 0.0011). (**C**) Western blot analysis of non-reducing SDS-PAGE of 15 μM GAPR-1, C32S, C63S or C32S/C63S incubated with 37.5 μM heparin and 20 μM Cu^2+^ at 37 °C for the indicated times (0, 3 and 18 h). Results were analyzed with C-terminal GAPR-1 antibody. (**D**) BN-PAGE analysis of GAPR-1 cysteine mutants in the absence and presence of metal ions. 2 μg C32S, C63S or C32S/C63S was incubated with or without 100 μM Zn^2+^ or Cu^2+^ at room temperature for 5 min.

We assessed the oxidation state of the cysteines in WT GAPR-1 by incubation with PEG-maleimide (~5 kDa), capable of reacting with reduced cysteines. SDS-PAGE analysis shows that for the majority of WT GAPR-1 both cysteines were labeled and that no unlabeled protein was detectable (Fig. 4A). To investigate the role of cysteine residues in copper-mediated amyloid-like aggregation in more detail, GAPR-1 mutants were constructed with one (C32S or C63S) or both cysteines (C32S and C63S) mutated to serine. In both GAPR-1 single cysteine mutants, almost complete labeling of the single cysteine with PEG-maleimide was observed, while C32S/C63S remained unlabeled, as expected (Fig. 4A). This shows that for all proteins the cysteines are predominantly in the reduced form. Of note: pre-incubation with NEM completely inhibited subsequent labeling with PEG-maleimide (Fig. 4A), confirming that inhibition of amyloid-like structures by NEM (Fig. 3A) is the result of efficient labeling of both cysteines in GAPR-1 with NEM. All three mutants showed a dramatic inhibition of ThT enhancement. In the presence of 20 µM Cu^2+^, both C32S and C32S/C63S did not show any substantial increase over 20 h, whereas for C63S ThT fluorescence only slightly increased as compared to WT GAPR-1 (Fig. 4B, left panel). The end point of ThT fluorescence (20 h) illustrates the significant differences between WT GAPR-1 and the three cysteine mutants (Fig. 4B, right panel). The inability of the cysteine mutants to form HMW structures was confirmed by Western blot analysis under non-reducing conditions. WT GAPR-1, C32S, C63S or C32S/C63S were incubated at 37 °C with heparin and 20 μM Cu^2+^ and aliquots were taken at 0 h, 3 h and 18 h, respectively. For WT GAPR-1 an increasing amount of structures was detected over time (Fig. 4C). However, only monomers and dimers were observed when one of the two cysteines was mutated. Only monomers were detected in the double cysteine mutant. These results imply that both cysteine residues play a role in the Cu^2+^-induced amyloid-like aggregation of GAPR-1.

The oligomeric state of each of the cysteine mutants was determined by BN-PAGE. C32S migrated similar to WT GAPR-1, i.e. ranging from dimer to tetramer, whereas both C63S and C32S/C63S were predominantly monomeric (Fig. 4D). In the presence of Cu^2+^, the monomeric content of C32S increased (Fig. 4D). Both cysteines in GAPR-1 are not in close proximity of the GAPR-1 dimer interface and therefore it is not clear how these cysteines affect the monomer-oligomeric status of GAPR-1. These results do imply, however, a role of GAPR-1 cysteines both in disulfide bond formation and in affecting the monomeric/oligomeric status of GAPR-1.

### Disulfide-bonded oligomers serve as nuclei

**Figure 5:**
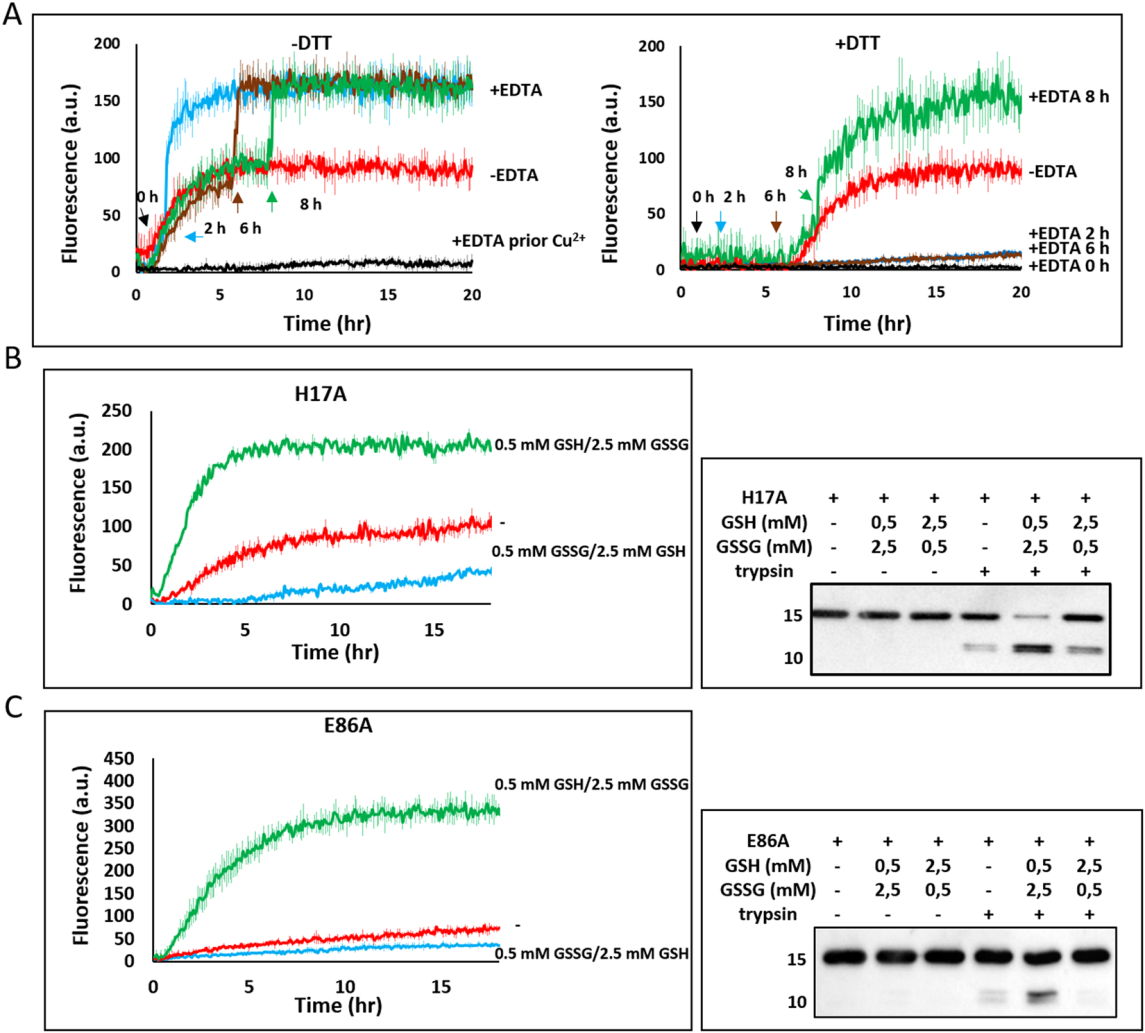
Disulfide-bonded oligomers serve as nuclei. (**A**) Kinetics of ThT fluorescence enhancement of GAPR-1 incubated with heparin and Cu^2+^ under non-reducing (left panel) and reducing (right panel) conditions. 15 μM GAPR-1 was incubated with 37.5 μM heparin and 100 μM Cu^2+^ in the absence and presence of 1 mM DTT at 37 °C, with addition of 1 mM EDTA at different timepoints (0 h, 2 h, 6 h, and 8 h). (**B**,**C**) The left panel shows kinetics of ThT fluorescence enhancement of 15 μM H17A (**B**) or E86A (**C**) incubated with 37.5 μM heparin at 37 °C under reducing (0.5 mM GSSG/2.5 mM GSH) and oxidative (0.5 mM GSH/2.5 mM GSSG) conditions, respectively. The right panels represent the Western blot analysis of limited trypsin digestion of H17A/E86A under reducing (0.5 mM GSSG/2.5 mM GSH) and oxidative (0.5 mM GSH/2.5 mM GSSG) conditions using N-terminal GAPR-1 antibody. The kinetics are the result of three independent experiments and the results represent the means (line in bold) +/− S.D.

Next, we assessed the role of copper in disulfide bond formation during the different stages (initiation and elongation/maturation) of GAPR-1 amyloid-like aggregation. To this end, EDTA was added at different time points during the aggregation process to remove the copper ions. Under non-reducing conditions, GAPR-1 rapidly forms amyloid-like fibers and as expected, addition of EDTA at the onset of the reaction fully inhibited aggregation (Fig. 5A, left panel). Surprisingly, addition of EDTA at different time points during aggregation did not halt the process but resulted in an immediate and steep rise in ThT fluorescence, eventually reaching a maximum value nearly twice as high as in the continuous presence of Cu^2+^ (Fig. 5A, left panel). These results suggest that Cu^2+^ is not required during the elongation/maturation phase but is required at the onset of the reaction. To investigate this in more detail, we repeated the experiment in the presence of DTT, causing a delay in the initiation phase (see Fig. 3B). Under these conditions, addition of EDTA at different time points during the lag-phase strongly inhibited the enhancement of ThT fluorescence during 20 h (Fig. 5A, right panel). In contrast, addition of EDTA at 8 h, when ThT fluorescence had commenced to increase, caused again a rapid jump in fluorescence (Fig. 5A, right panel). These results confirm that although copper binding is crucial for initiation of GAPR-1 fibrillation, it is not required for its continuation. During the elongation/maturation phase, amyloid-like aggregation is less sensitive to oxidizing/reducing conditions and proceeds more efficiently in the absence of Cu^2+^ ions.

Together, our results indicate a role for Cu^2+^ and redox balance during the initiation phase of GAPR-1 amyloid-like aggregation. To determine whether Cu^2+^ directly affects the redox balance, the assay was repeated using monomeric GAPR-1 mutants E86A and H17A that form amyloid-like aggregates in the absence of Cu^2+^. These GAPR-1 mutants were incubated with heparin in buffer containing different ratios of reduced and oxidized glutathione (GSH and GSSG, respectively). H17A and E86A in the presence of heparin already caused a modest increase in ThT fluorescence (Fig. 5B,C, left panel). For both mutants, ThT fluorescence increased dramatically under oxidative condition (0.5 mM GSH/2.5 mM GSSG). However, under reduced condition (2.5 mM GSH/0.5 mM GSSG), the increase of ThT fluorescence was inhibited (Fig. 5B,C, left panel). Trypsin digestion assay shows that for both H17A and E86A mutant proteins, proteolysis was also enhanced under oxidative conditions (Fig. 5B,C, right panel), confirming structural changes upon cysteine oxidation.

### Comparison with zinc-mediated GAPR-1 amyloid-like aggregation

**Figure 6:**
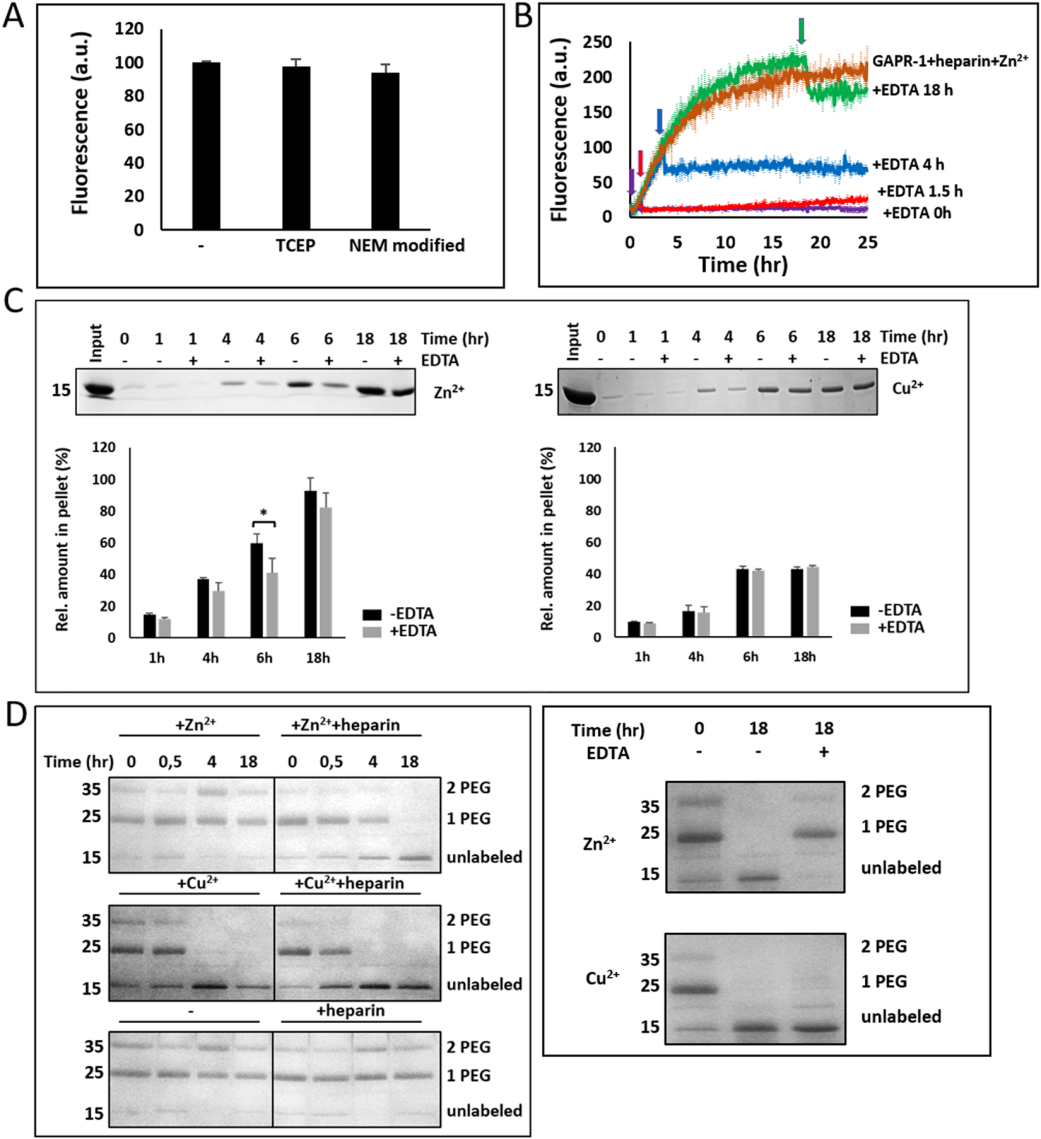
Comparison with zinc-mediated GAPR-1 amyloid-like aggregation. (**A**) Zinc-dependent ThT fluorescence enhancement of GAPR-1 under reducing conditions or with modified cysteines. 15 μM GAPR-1, either in the presence of 1 mM TCEP or with NEM-modified cysteines, was incubated with 37.5 μM heparin and 100 μM Zn^2+^ for 20 h and ThT fluorescence intensity is shown relative to control conditions (without TCEP and NEM modification). The results represent the means (+/− S.D.) of three independent experiments. (**B**) Kinetics of ThT fluorescence enhancement of 15 μM GAPR-1 incubated with 37.5 μM heparin and 100 μM Zn^2+^ at 37 °C, with addition of 1 mM EDTA at different timepoints (0 h, 1.5 h, 4 h, and 18 h). The results represent the means (line in bold +/− S.D.) of three independent experiments. (**C**) 15 μM GAPR-1 was pre-incubated with 100 μM Zn^2+^ (left panel) or 20 μM Cu^2+^ (right panel) in the presence of 37.5 μM heparin at 37 °C for 1 h, 4 h, 6 h and 18 h, respectively, followed by incubation with or without 2 mM EDTA at room temperature for 30 min. Following centrifugation at 14,000 g for 30 min, the pellet fraction was analyzed by SDS-PAGE with Coomassie blue staining. The amount of GAPR-1 in the pellet is expressed as a percentage of the total input as determined by densitometry. The results represent the means (+/− S.D.) of three independent experiments. Stars indicate the statistical significance using t-test (two-sample assuming equal variances): *0.01 < P-value = 0.0286 < 0.05. (**D**) 15 μM GAPR-1 was incubated with or without 100 μM Zn^2+^or 20 μM Cu^2+^ in the absence or presence of 37.5 μM heparin at 37 °C for 0 h, 0.5 h, 4 h and 18 h. This was followed by incubation with 1 mM PEG-maleimide at room temperature for 1 h and analysis by SDS-PAGE with Coomassie blue staining (left panel). 15 μM GAPR-1 was incubated with 100 μM Zn^2+^ or 20 μM Cu^2+^ and 37.5 μM heparin at 37 °C for 18 h and subsequently incubated with or without 2 mM EDTA at room temperature for 30 min. PEG labeling was analyzed by SDS-PAGE with Coomassie blue staining (right panel).

The differential effects of Cu^2+^ and Zn^2+^ on amyloid-like aggregation of GAPR-1 mutants H54A and H103A suggest that the fibrillation pathway for both metal ions is mechanistically distinct. Indeed, Zn^2+^-induced enhancement of ThT fluorescence was not significantly affected for GAPR-1 with NEM-modified cysteines (Fig. 6A), indicating that both cysteines do not play a role in this process. In agreement, we found no difference in the zinc-dependent aggregation pathway of GAPR-1 under control and reducing (TCEP) conditions, suggesting that disulfide bond formation does not play a role in the zinc-dependent pathway neither.

The requirement for zinc ions during the distinct stages of fibrillation was also investigated. Addition of EDTA during the fibrillation process (0 h, 1.5 h, 4 h and 18 h, respectively) caused an immediate stop in amyloid-like aggregation in all cases, accompanied by an immediate small reduction in ThT fluorescence (Fig. 6B). This suggests that the continuous presence of zinc is required for aggregation to proceed and that upon removal of zinc ions the ThT-positive HMW structures formed partially disintegrate. Sedimentation analysis was used to confirm this. GAPR-1 was incubated with heparin and Zn^2+^ and EDTA was added at different timepoints during fibrillation. Subsequently, HMW structures were sedimented by centrifugation. Figure 6C (left panel) shows that removal of zinc ions at any timepoint notably decreased the amount of sedimented protein. In contrast, removal of Cu^2+^ ions did not decrease the amount of sedimented protein (Fig. 6C, right panel).

The results indicate that Zn^2+^-mediated protein aggregation can at least be partially reversed. To investigate this, we used PEG labeling to monitor the accessibility of GAPR-1 cysteines during Zn^2+^-induced aggregation. GAPR-1 was incubated under various conditions and after taking aliquots at different times points, the samples were labeled with PEG-maleimide. GAPR-1 incubated with Zn^2+^ and heparin showed progressively fewer accessible cysteines over time (Fig. 6D, left panel). We then assessed the reversibility of this process by investigating cysteine accessibility following the removal of zinc ions from HMW structures. To this end, GAPR-1 was first incubated with heparin and Zn^2+^ for 18 h, after which EDTA was added. Following brief incubation, PEG-maleimide was added to label the accessible cysteines. Figure 6D (right panel) shows that the cysteines that were protected from PEG modification in the zinc-aggregates, became highly accessible again after addition of EDTA. This shows that cysteine residues in the zinc-induced GAPR-1 aggregates are shielded from modification in a reversible manner. In marked contrast, the presence of Cu^2+^, both with or without heparin, inhibited labeling of cysteines as expected through oxidation (Fig. 6D, left panel). In addition, cysteine inaccessibility was not reversible in Cu^2+^-induced GAPR-1 aggregates (Fig. 6D, right panel).

## Discussion

GAPR-1 exists in various oligomeric forms. It is present as dimers on Golgi membranes and displays amyloidogenic properties *in vitro* that can be regulated by Zn^2+^ binding^17,20,24^. This implies that the mechanisms underlying self-interaction are important for the functionality of GAPR-1 in autophagy and other cellular pathways^13,20^. We have shown here that formation of GAPR-1 amyloid-like structures can occur through distinct molecular pathways dependent on zinc/copper ion binding and redox conditions, providing novel insights into the possible regulatory mechanisms controlling GAPR-1 oligomerization and aggregation.

Using BN-PAGE analysis we show that the native quaternary structure of GAPR-1 is modulated by zinc and copper ions. Recombinant WT GAPR-1 is mainly multimeric (ranging from dimer to tetramer) in solution. Binding of Cu^2+^ causes an immediate dissociation of multimeric into monomeric GAPR-1. In the monomeric state, aggregation-prone regions (APRs) that are hidden in the multimeric state become exposed, resulting in efficient aggregation. This could be due to increased conformational flexibility in the monomer and/or because they were buried in the dimer interface. The exposure of APRs is corroborated by two GAPR-1 mutants, H17A and E86A, that are predominantly monomeric in solution. Incubation of these mutants with heparin caused increased ThT fluorescence without the addition of metal ions. Nonetheless, the presence of either Zn^2+^ or Cu^2+^ additionally enhanced both ThT fluorescence, suggesting that both metal ions stabilize conformations that amplify the amyloidogenic propensity of GAPR-1.

Destabilization of native state multimers is a common mechanism proposed for several other amyloidogenic proteins^27–32^. A well-known example is transthyretin (TTR), which commonly exists as a homotetramer. Various factors such as mutation, oxidation, pH and ligand binding impact the stability of TTR, with dissociation into monomers consequently resulting in pathogenetic fibrillation^27,28,33–35^. The yeast pyruvate kinase Cdc19 uses similar mechanisms to form functional amyloid^29,30,36^. We now show that metal ions differentially affect the monomeric and oligomeric status of GAPR-1. Whereas Cu^2+^ ions cause an immediate shift from multimeric to monomeric GAPR-1, Zn^2+^ ions cause a similar shift, but only after prolonged incubation. For numerous proteins involved in amyloid-related neurodegenerative diseases, copper and zinc homeostasis plays crucial roles^22,23,37^. A well-studied example is superoxide dismutase 1 (SOD1), to which Zn^2+^ and Cu^2+^ binding is required for structural stability and proper function. Either demetallation or aberrant metal binding has been shown to promote protein misfolding and pathogenic aggregation^23,38,39^. Here we have shown that zinc and copper ions differentially affect the structural regulation of GAPR-1 and speculate that the distinct GAPR-1 oligomeric or amyloid-like structures serve specific physiological purposes. In line with this, many functional amyloids have recently been discovered as a novel physiological mechanism to regulate a variety of cellular activities, including storage of hormone peptides^40,41^, fertilization^42–44^, necroptosis^45^, pigmentation^46^, antimicrobial responses^47^ and adaptation to stress conditions^29,48^. Moreover, some functional aggregates are fully reversible in a highly regulated manner^28,29,41^.

The zinc-induced GAPR-1 amyloid-like aggregation is different from Cu^2+^-induced aggregation pathway in many ways. Most importantly, Zn^2+^ binding to the putative CAP metal-binding site causes a slow shift to monomeric GAPR-1 species, which is independent of the redox conditions and requires the continuous presence of zinc. In addition, the amyloid-like structures formed in the presence of Zn^2+^ have a different structure as shown by TEM and the fact that cysteines in Zn^2+^-induced GAPR-1 aggregates are protected from modification in a reversible manner illustrates the reversible nature of Zn^2+^-induced amyloid-like fibrils. Zn^2+^ coordination to cysteines is a known mechanism to protect free thiol groups from oxidation^49,50^. However, Zn^2+^-induced amyloid-like aggregation still occurs in the GAPR-1 mutant without cysteines. We therefore consider it more likely that the two cysteine residues become buried in the amyloid-like structures, making them unavailable for modification. In this respect, it is interesting to note that in the GAPR-1 crystal structure both cysteines are surface-exposed and highly oxidized. In contrast, in the IP6-bound structure of GAPR-1, Cys63 is not oxidized and its side chain is oriented inwards^18^. Thus, different orientations or exposures of the cysteines in GAPR-1 can have drastic effects on the redox potential of these amino acids. Protection of cysteines could be an essential mechanism for the generation of reversible amyloid aggregates in the presence of Zn^2+^ and to protect these aggregates from becoming irreversible as observed in the Cu^2+^-induced amyloid fibrils (in which cysteines are accessible for modification).

**Figure 7:**
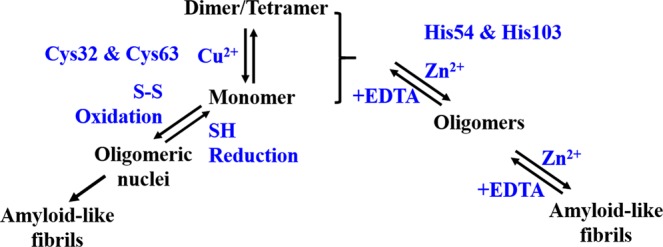
Model of distinct Zn^2+^- and Cu^2+^-induced amyloid-like aggregation pathways of GAPR-1 in the presence of heparin. In the Cu^2+^-induced pathway, GAPR-1 quaternary structure is modulated by Cu^2+^ binding, shifting native multimers to monomers. Redox-dependent disulfide bond formation is crucial during the initiation when oligomeric nuclei are formed. In the Zn^2+^-dependent pathway, reversible amyloid-like aggregates are formed. Cys32 and Cys63 play essential roles in the Cu^2+^ induced GAPR-1 aggregation pathway, while His54 and His103 are important in the Zn^2+^ induced aggregation pathway.

Cu^2+^-induced GAPR-1 amyloid-like aggregation does not involve the conserved metal binding site. This was surprising as in the crystal structures of other CAP proteins, several different transition metals, including Cu^2+^, localize to this binding site^2–7^. The copper-induced pathway is modulated by the thiol/disulfide oxidation status of the two cysteines in GAPR-1. This pathway involves formation of copper-catalyzed disulfide-linked oligomeric species that subsequently serve as nuclei for typical amyloid-related fibrils. We show that the redox environment can either inhibit or stimulate the formation of GAPR-1 fibrils. Under reducing conditions, copper ions are required to catalyze formation of oligomeric nuclei, which is the rate limiting step as shown by ThT fluorescence kinetic measurements. A model for the distinct pathways is displayed in Fig. 7.

GAPR-1 is mainly localized at the cytosolic side of Golgi membrane^15^, where cysteines tend to remain in the reduced state^51^. Hence, the cytosolic orientation makes the involvement of disulfide bonds in GAPR-1 oligomerization less likely to occur. However, neighboring positively charged residues, such as the lysine adjacent to Cys32 of GAPR-1, lower the pK_a_ value of its thiol group^52^ favoring the thiolate form, which is very reactive towards oxidants and thiols^53^. Cu^2+^ is a redox-active metal ion which is able to catalyze formation of disulfides^54^. Disulfide bridges are dynamic in response to the changes of redox environment in the cell^53^ and often play critical roles in amyloid formation^45,55–57^.

Are these properties reflected in other members of the CAP superfamily? At the molecular level, one of the cysteine residues of GAPR-1 (Cys63) is semi-conserved within the superfamily while Cys32 is non-conserved. Compared to GAPR-1, other CAP superfamily members generally contain more cysteine residues including in their CAP domain, most of which are involved in disulfide bridges. These are thought to convey structural stability in the extracellular environment, where most CAP family members reside. While the importance of redox buffers (*e.g.* glutathione, cysteine, thioredoxin) for maintaining a reducing intracellular environment is well established, the extracellular space is commonly regarded as an oxidizing redox-inert microenvironment. However, emerging evidence shows that the extracellular redox environment is dynamic, carefully regulated and intimately coupled to intracellular metabolism^58,59^. Likewise, extracellular zinc and copper levels are tightly interwoven with the regulation of cellular signaling pathways^60,61^. As many CAP proteins form functional oligomers *e.g.* during fertilization and immune regulation^4,7,25,62,63^, the metal ion and redox balance may provide important means to regulate their activity.

## Methods

### Reagents

Heparin was purchased from Santa Cruz Biotechnology (Heidelberg, Germany). Trypsin, DpnI, Phusion DNA Polymerases were obtained from Thermo Fisher Scientific (Eindhoven, the Netherlands). Thioflavin T, ZnCl_2,_ CuCl_2_, MgCl_2_·6H_2_O, CaCl_2_·2H_2_O, FeCl_3_, ethylenediaminetetraacetic acid (EDTA), tris(2-carboxyethyl)phosphine hydrochloride (TCEP), β-mercaptoethanol (BME), dithiothreitol (DTT), N-Ethylmaleimide (NEM), methoxypolyethylene glycol maleimide (PEG-maleimide, 5 kDa), reduced and oxidized glutathione (GSH and GSSG) were from Sigma-Aldrich (St. Louis, USA).

### Plasmids

pQE60-GAPR-1 WT plasmid was described before^17,64^. pQE60-GAPR-1 H17A, E86A, C32S, C63S and C32S/C63S mutants were generated by site-directed mutagenesis using the following mutagenic primers. Altered sequences are shown in bold in Table 1.

**Table 1:** Primers used for site-directed mutagenesis.

GAPR-1 mutation	Forward	Reverse
H17A	5′-gtcctgaaggcc**gcc**aatgagtaccg-3′	5′-cggtactcatt**ggc**cggccttcaggac-3′
E86A	5′-ggtacagt**gca**atcaagaactataacttc-3′	5′-gttcttgat**tgc**actgtaccatctatcagc-3′
C32S	5′-cactgaagctc**tcc**aagaacctcaaccg-3′	5′-ggttctt**gga**gagcttcagtgggggac-3′
C63S	5-cgtggccag**tct**ggggagaacctgcatg-3′	5′-gttctcccc**aga**ctggccacggctggac-3′

Mutations in GAPR-1 were verified by DNA sequencing (BaseClear, Leiden, the Netherlands). The protocol used to express and purify GAPR-1 mutants was the same as described for WT GAPR-1^17,64^.

### Thioflavin T fluorescence assay

The kinetics of GAPR-1 amyloid-like aggregation in the presence of heparin and different metal ions were monitored by Thioflavin T (ThT) fluorescence. Reaction mixtures contained 15 μM GAPR-1, 37.5 μM heparin and 50 μM ThT with 0–1,000 μM Zn^2+^, Cu^2+^, Ca^2+^, Mg^2+^ and Fe^3+^, respectively, in NT-50 buffer (25 mM Tris, 50 mM NaCl, pH 7.4), and were incubated in sealed 96-well plates (Flat Clear Bottom Black Polystyrene, Corning, USA) at 37 °C for 20 h. Fluorescence was measured in a CLARIOstar microplate reader (BMG LABTECH, Germany). ThT fluorescence emission was recorded at 488 nm after excitation at 449 nm with agitation before every measurement. The results represent the means (+/− S.D.) of three independent experiments.

### Transmission electron microscopy (TEM)

15 μM GAPR-1 was incubated with 37.5 μM heparin and 100 μM Cu^2+^ at 37 °C for 3 h and 24 h. 10 µl of each sample was placed on a 100 mesh glow discharged gold grid (Quantifoil Micro Tools GmbH, Jena,Germany). After 30 sec, excess fluid was removed, and the grid was washed twice with ddH_2_O for 30 sec each time. The grid was then negatively stained with 2% uranyl acetate in ddH_2_O for twice, 15 sec and 30 sec, respectively. After removal of excess fluid, the grid was dried on air. Samples were viewed in Tecnai 20 LaB6 transmission electron microscope (FEI, Eindhoven, the Netherlands) at 200 kV. Images were recorded using a 4 K square pixelEagle CCD camera (FEI, Eindhoven, the Netherlands).

### Circular dichroism (CD) spectroscopy

CD spectra of GAPR-1 were measured on a JASCO J-810 spectropolarimeter (Jasco Co. Ltd., Tokyo, Japan) at 37 °C as described by Jie *et al*.^24^. Briefly, CD spectra of 15 μM GAPR-1 in the presence of 0–100 μM Cu^2+^ in NT-50 buffer were acquired using a quartz cuvette (1 mm path length). Measurements were performed by scanning from 260 to 190 nm and repeated four times.

### Trypsin digestion

30 μM GAPR-1 was incubated with increasing Cu^2+^ concentrations (0–500 μM) in a total volume of 20 μl NT-50 buffer at 37 °C for 30 min, after which trypsin was added in a molar ratio of 1:50 (trypsin: GAPR-1) and incubated at 37 °C for 30 min. The reaction was terminated by addition of Laemmli sample buffer. Protein samples were analyzed by SDS-PAGE and Western blot using five different peptide antibodies (Ab 1–5) that are directed against different epitopes in GAPR-1. The epitopes and antibodies are described by Jie *et al*.^24^.

### Blue native PAGE

Protein samples (2 μg) were mixed with 4x native sample buffer (200 mM Tris, 40% (v/v) glycerol, 0.08% (w/v) Coomassie Blue G-250 (Sigma-Aldrich, St. Louis, USA), pH 6.8) and loaded onto a 4–20% Mini-PROTEAN TGX Precast gel (Bio-Rad, California, USA). Electrophoresis was carried out using anode buffer (25 mM Tris, 192 mM glycine, pH 7.0), and blue cathode buffer (25 mM Tris, 192 mM glycine, 0.02% (w/v) Coomassie G-250, pH 7.0)^65^ at 4 °C for 1 h at 100 V.

### SDS-PAGE and western blot

Formation of high molecular weight (HMW) structures of WT GAPR-1 and cysteine mutants was analyzed by Western blot. 15 μM WT GAPR-1, C32S GAPR-1, C63S GAPR-1 or C32S/C63S GAPR-1 was incubated with 37.5 μM heparin and 20 μM Cu^2+^ in NT-50 buffer at 37 °C. Aliquots taken at different timepoints (0 h, 3 h and 18 h) were treated with Laemmli sample buffer in the presence or absence of β-mercaptoethanol (BME), separated on SDS-PAGE gel and analyzed by Western blot using a C-terminal GAPR-1 antibody^15^.

### N-Ethylmaleimide (NEM) labeling assay

60 μM GAPR-1 was incubated with 10 mM NEM at room temperature for 1 h in the dark. Unreacted NEM was removed by three repetitive steps of dilution in NT-50 buffer and concentration using Amicon 10 kDa MWCO centrifugal filter (Merck, Darmstadt, Germany). Protein concentration was determined using Pierce Coomassie (Bradford) assay kit (Thermo Fisher, Eindhoven, the Netherlands) and the degree of NEM-modification was determined by subsequent PEG-maleimide labelling and SDS-PAGE.

### PEG-maleimide labelling assay

15 μM GAPR-1 was incubated with 100 μM Zn^2+^ or 20 μM Cu^2+^ in the absence or presence of 37.5 μM heparin at 37 °C. Aliquots taken at different timepoints (0, 0.5, 4 and 18 h) were incubated with 1 mM PEG-maleimide at room temperature for 1 h and analyzed by SDS-PAGE with Coomassie blue staining.

### Sedimentation analysis

15 μM GAPR-1 was incubated with 100 μM Zn^2+^ or 20 μM Cu^2+^ and 37.5 μM heparin at 37 °C. 2 mM EDTA was added after 1, 4, 6 and 18 h, respectively, and incubated for an additional 30 min at room temperature, followed by centrifugation at 14,000 g for 30 min. Protein content in the pellet fraction was analyzed by SDS-PAGE with Coomassie blue staining and quantified using densitometry (Image Lab, Bio-Rad, California, USA). The results represent the mean (+/− S.D.) of three independent experiments.

## Supplementary information

Supplementary Information.

Supplementary Figure.
