# Supplementary material for: Metal ions and redox balance regulate distinct amyloid-like aggregation pathways of GAPR-1
Source: Sci Rep. 2019 Oct 21;9:15048. doi: 10.1038/s41598-019-51232-7 (PMC6803662; doi:10.1038/s41598-019-51232-7)

Supplementary information

Metal ions and redox balance regulate distinct amyloid-like aggregation pathways of GAPR-1

Jie Sheng[§], Nick K. Olrichs[§], Willie J. Geerts[#], Dora V. Kaloyanova[§], J. Bernd Helms[§]*

[§] Department of Biochemistry and Cell Biology, Faculty of Veterinary Medicine, Utrecht University, Utrecht, the Netherlands

[#] Biomolecular Imaging, Bijvoet Center, Utrecht University, Utrecht, the Netherlands.

* To whom correspondence should be addressed: J.B.H. (J.B.Helms@uu.nl)

Supplementary figure legends

Supplementary Figure 1. Cysteines are essential in Cu^{2+}-induced GAPR-1 amyloid-like aggregation. 15 μM WT GAPR-1 with or without NEM-modified cysteines was incubated with 37.5 μM heparin and 20 μM Cu^{2+} at 37 °C. Aliquots taken from 0 h, 3 h and 18 h were analyzed by non-reducing SDS-PAGE and Western blot using a C-terminal GAPR-1 antibody.

Supplementary Figure 2. Uncropped Western blots for Figure 2B and 2E, and uncropped BN-PAGE gels for Figure 2C. Dashed boxes indicate cropped areas.

Supplementary Figure 3. Uncropped Western blot for Figure 3D. Dashed box indicates the cropped area.

Supplementary Figure 4. Uncropped Coomassie blue-stained SDS-PAGE gel for Figure 4A, uncropped Western blots for Figure 4C, and uncropped BN-PAGE gels for Figure 4D. Dashed boxes indicate cropped areas.

Supplementary Figure 5. Uncropped Western blots for Figure 2B and 2C. Dashed boxes indicate cropped areas.

Supplementary Figure 6. Uncropped Coomassie blue-stained SDS-PAGE gel for Figure 6C and 6D. Dashed boxes indicate cropped areas.

Supplementary Figure 7. Uncropped Western blot for Supplementary Figure 1. Dashed box indicates the cropped area.

Supplementary Figure 1

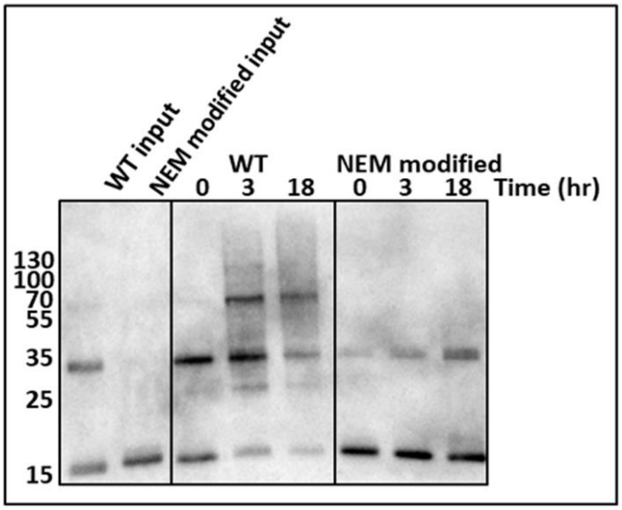

Supplementary Figure 2

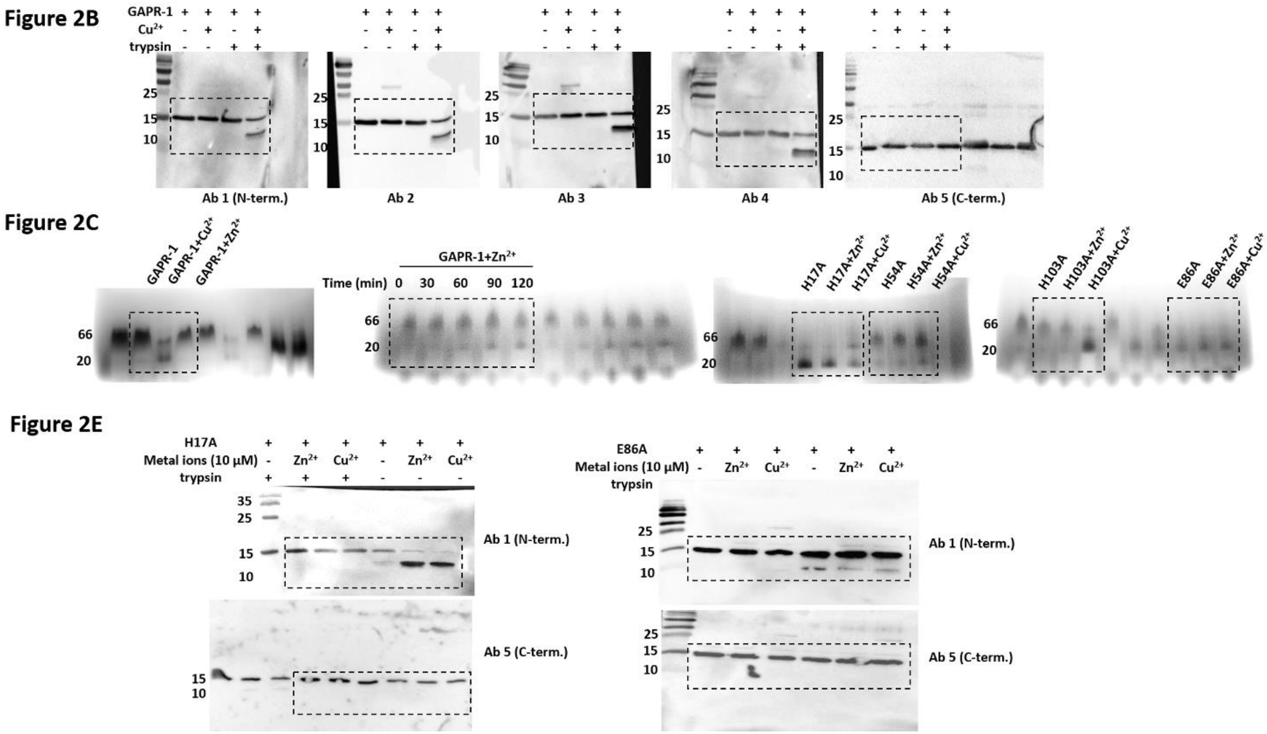

Supplementary Figure 3

Figure 3D

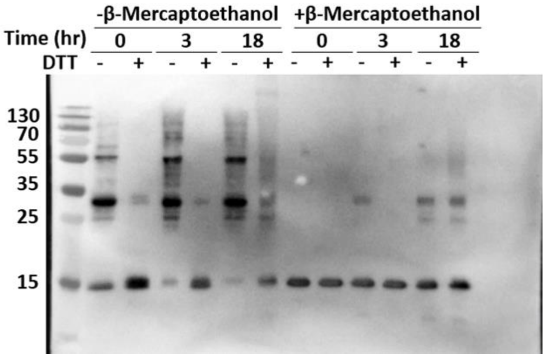

Supplementary Figure 4

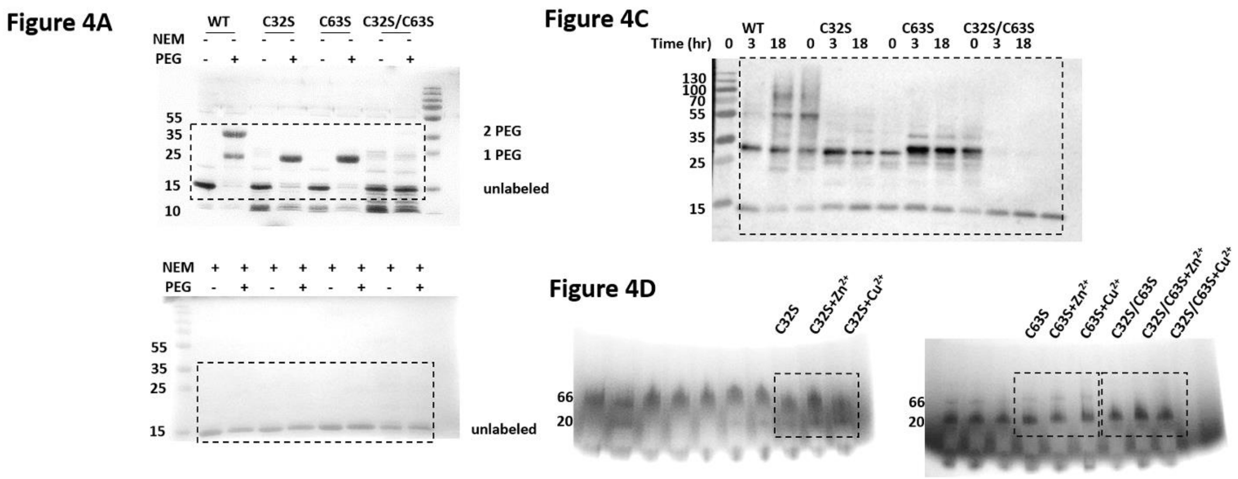

Supplementary Figure 5

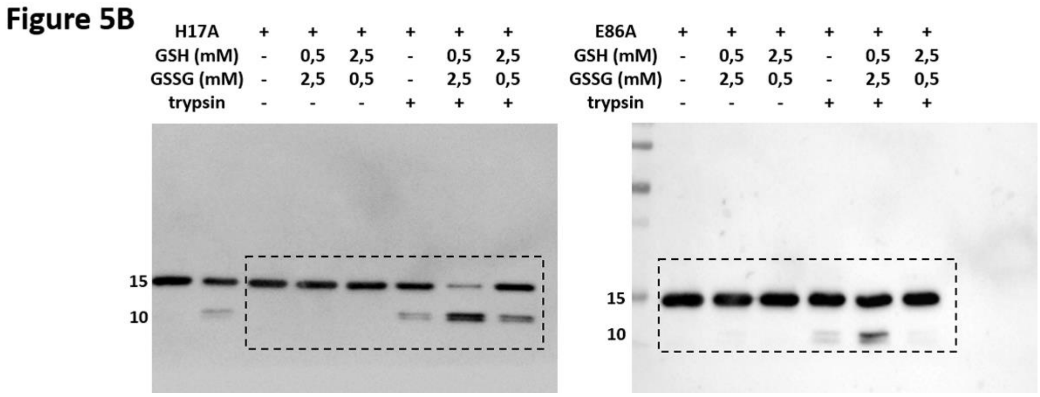

Supplementary Figure 6

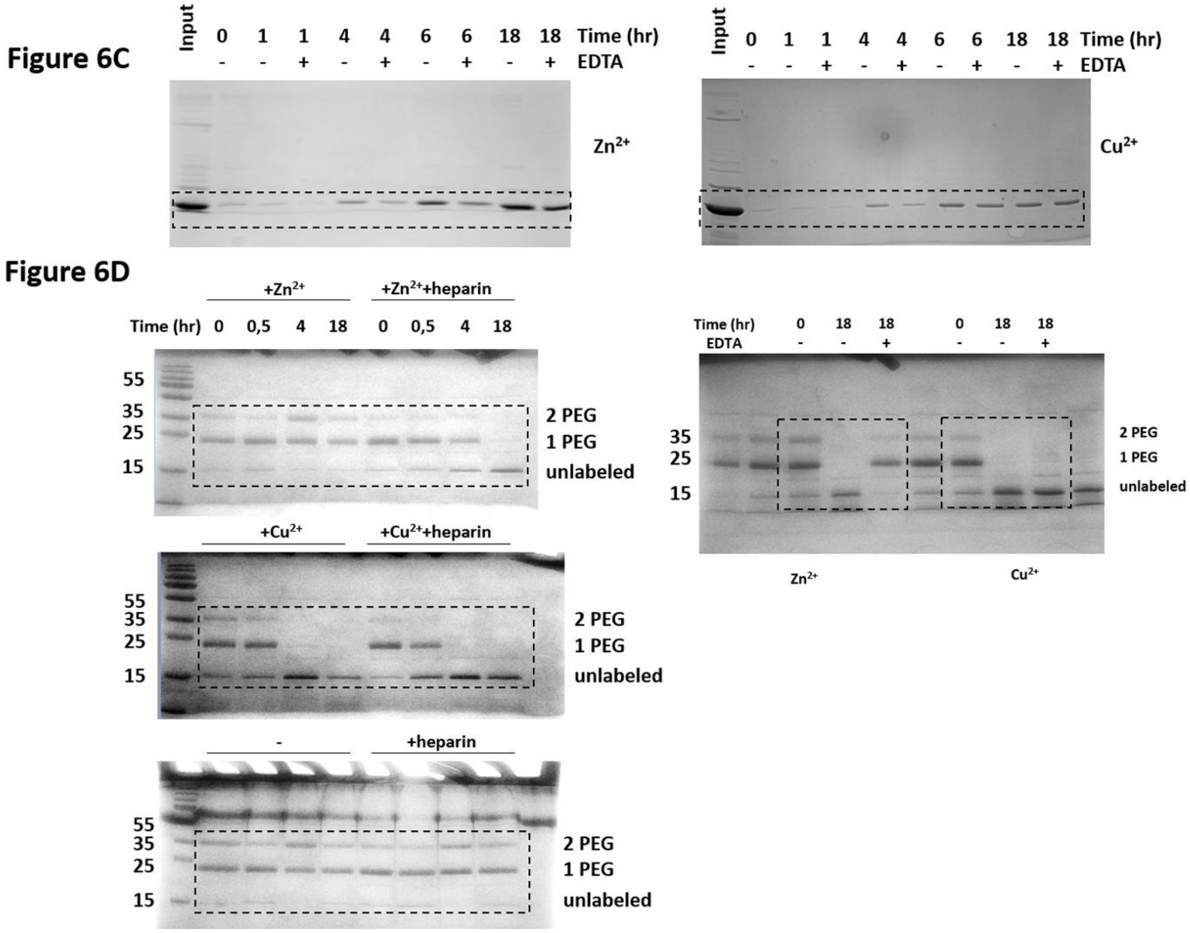

Supplementary Figure 7

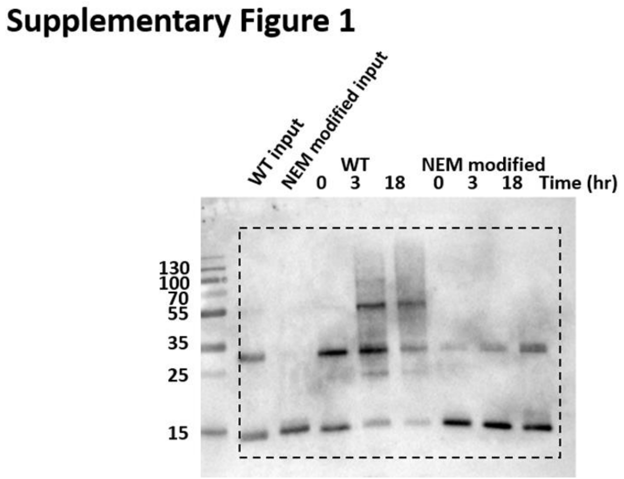

Supplement: Supplementary file 1 — Supplementary Information. [file 41598_2019_51232_MOESM1_ESM.pdf]
