# Supplementary figures and images for: Metal ions and redox balance regulate distinct amyloid-like aggregation pathways of GAPR-1
Source: Sci Rep. 2019 Oct 21;9:15048. doi: 10.1038/s41598-019-51232-7 (PMC6803662; doi:10.1038/s41598-019-51232-7)

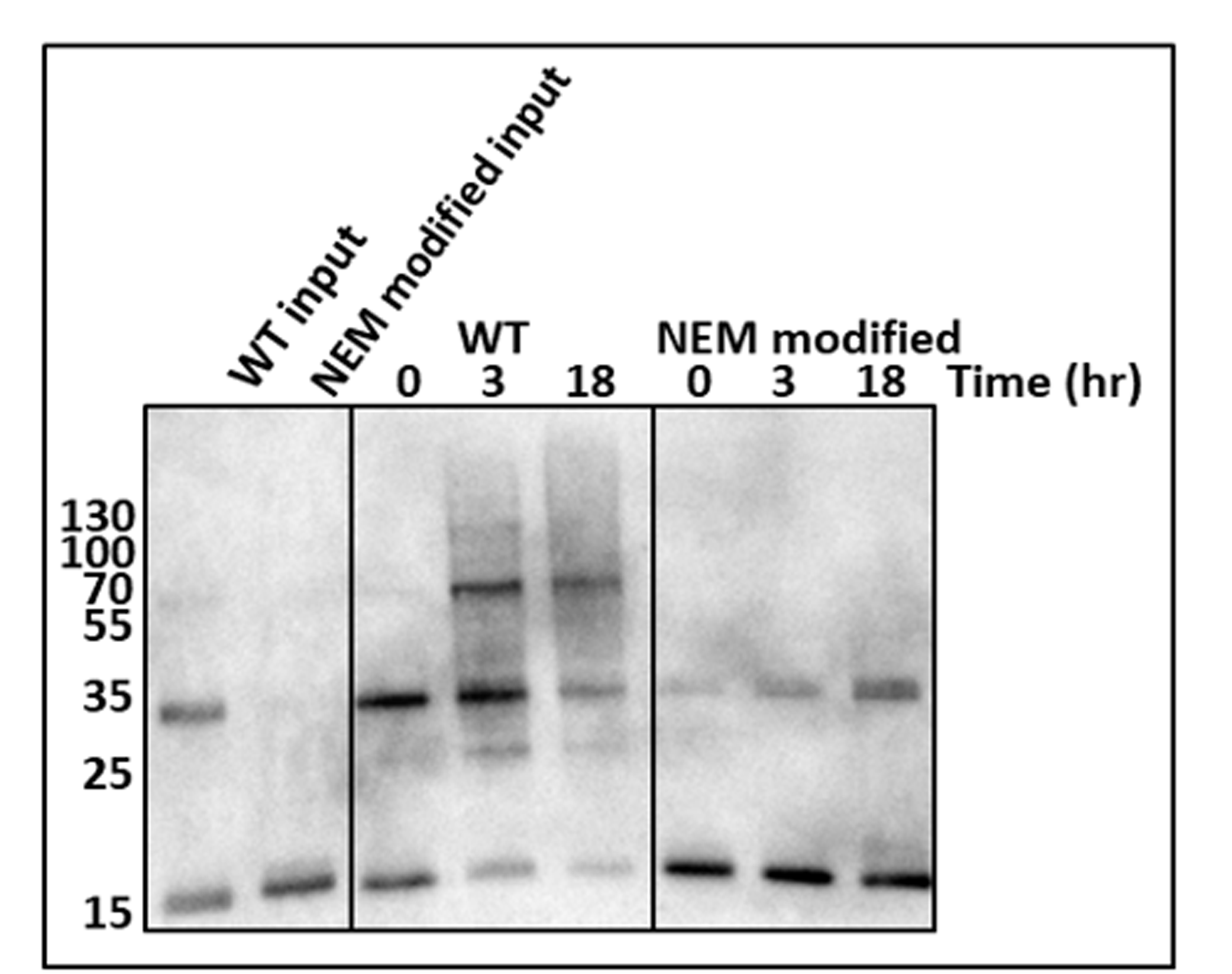

Supplement: Supplementary file 2 — Supplementary Figure. [file 41598_2019_51232_MOESM2_ESM.tif]
